# Design and Characterization of a Novel Core–Shell Nano Delivery System Based on Zein and Carboxymethylated Short-Chain Amylose for Encapsulation of Curcumin

**DOI:** 10.3390/foods13121837

**Published:** 2024-06-11

**Authors:** Zhiwei Lin, Linjie Zhan, Kaili Qin, Yang Li, Yang Qin, Lu Yang, Qingjie Sun, Na Ji, Fengwei Xie

**Affiliations:** 1 College of Food Science and Engineering, Qingdao Agricultural University, Qingdao 266109, China; lzwhappy2021@163.com (Z.L.); zlj980606@163.com (L.Z.); liyangstrel@qau.edu.cn (Y.L.); qinyjnu@163.com (Y.Q.); yanglusp@163.com (L.Y.); phdsun@163.com (Q.S.); 2 Qingdao Special Food Research Institute, Qingdao 266109, China; 3 School of Public Health, Anhui University of Science and Technology, Huainan 232001, China; 18306421320@163.com; 4 Department of Chemical Engineering, University of Bath, Bath BA2 7AY, UK; fwhsieh@gmail.com

**Keywords:** carboxymethylated short-chain amylose, core–shell nanoparticles, biopolymer molecular interactions, anti-solvent precipitation

## Abstract

Curcumin is a naturally occurring hydrophobic polyphenolic compound with a rapid metabolism, poor absorption, and low stability, which severely limits its bioavailability. Here, we employed a starch–protein-based nanoparticle approach to improve the curcumin bioavailability. This study focused on synthesizing nanoparticles with a zein “core” and a carboxymethylated short-chain amylose (CSA) “shell” through anti-solvent precipitation for delivering curcumin. The zein@CSA core–shell nanoparticles were extensively characterized for physicochemical properties, structural integrity, ionic stability, in vitro digestibility, and antioxidant activity. Fourier-transform infrared (FTIR) spectroscopy indicates nanoparticle formation through hydrogen-bonding, hydrophobic, and electrostatic interactions between zein and CSA. Zein@CSA core–shell nanoparticles exhibited enhanced stability in NaCl solution. At a zein-to-CSA ratio of 1:1.25, only 15.7% curcumin was released after 90 min of gastric digestion, and 66% was released in the intestine after 240 min, demonstrating a notable sustained release effect. Furthermore, these nanoparticles increased the scavenging capacity of the 1,1-diphenyl-2-picrylhydrazyl (DPPH•) free radical compared to those composed solely of zein and were essentially nontoxic to Caco-2 cells. This research offers valuable insights into curcumin encapsulation and delivery using zein@CSA core–shell nanoparticles.

## 1. Introduction

Curcumin, a natural polyphenolic compound found in the ginger family, is recognized for its antioxidant, anti-cancer, anti-inflammatory, and anti-aging properties [1]. Despite these benefits, its application in specific functional foods is hindered by challenges like poor physical and chemical stability, high hydrophobicity, and low bioavailability [2]. To overcome these challenges, easily fabricated nanoparticles with excellent biocompatibility become crucial for encapsulating and delivering bioactive substances. Natural polymeric nanoparticles, particularly those utilizing proteins and polysaccharides, have emerged as highly promising systems for enhancing curcumin delivery [3]. These systems, designed to encapsulate, protect, transport, and release functional substances like curcumin, capitalize on the natural biodegradability and structure-forming properties of proteins and polysaccharides. This approach effectively addresses issues of poor stability and low bioavailability [4,5].

Zein, a vital plant protein, finds broad applications across various fields, including food delivery systems [6], pharmaceuticals [7], and biomedicine [8]. Prior research has shown that amphiphilic zein can spontaneously form nanoparticles in response to solvents of increasing polarity [9]. However, due to its intrinsic hydrophobicity, zein nanoparticles tended to aggregate, resulting in larger aggregates and a significant increase in particle size. Additionally, zein nanoparticles are easily digested in the gastrointestinal tract, leading to the premature degradation of curcumin. Therefore, directly applying them in a curcumin delivery system could not achieve optimal results. To tackle these challenges, a range of hydrophilic polysaccharides have been explored to prevent zein nanoparticle aggregation and broaden their applications [10]. Examples include κ-carrageenan [11], pectin [12], alginate [13], hyaluronic acid [14], and soluble soybean polysaccharides [15], serving as effective natural stabilizers for nanoparticles. However, the high viscosity and weak charge of specific polysaccharides may impose practical limitations [16]. Hence, identifying additional natural polysaccharides capable of stabilizing zein particles remains a crucial research objective.

Carboxymethyl starch, an anionic starch derivative modified to enhance hydrophilicity, encounters practical limitations due to its substantial particle size, especially in nanoparticle preparation [17]. Short-chain amylose, a low-molecular-weight hydrophilic linear polymer obtained through the enzymatic debranching of amylopectin, finds widespread use in the pharmaceutical and health industries. Its application serves to augment the capabilities of carboxymethyl starch. Ji et al. demonstrated the use of carboxymethylated short-chain amylose in preparing insulin-loaded zein/carboxymethylated short-chain amylose (CSA) complex nanoparticles for oral insulin delivery [18]. Recent research has further showcased enhancements in the stability of nanoparticles prepared with carboxymethylated short-chain amylose [19].

The objective of this study was to fabricate zein-CSA “core-shell” nanoparticles for curcumin delivery, aiming to enhance the loading, stability, and release properties of curcumin. Firstly, due to the self-assembly characteristics of zein, the zein-containing curcumin could be formed as the “core” during the antisolvent process. Subsequently, CSA was adsorbed to the zein core as a “shell” due to the electrostatic interaction, forming a “core-shell” structure. The particle size, polydispersity index (PDI), zeta potential, encapsulation efficiency (EE), loading capacity (LC), controlled release kinetics, antioxidant potential, and cytotoxicity of curcumin-coated zein@CSA “core-shell” nanoparticles were investigated. The findings of this study might contribute to the assessment of the potential application of the nanoparticles as a promising protein–polysaccharide delivery system in fields such as drug delivery and functional foods.

## 2. Materials and Method

### 2.1. Materials

Waxy corn starch (~98% amylopectin) was supplied by Zhucheng Xingmao Co., Ltd. (Zhucheng, China). Pullulanase (4461.6 NPUN/g) was obtained from Beijing Novozymes Investment Co., Ltd. (Beijing, China). Zein was purchased from Sigma-Aldrich (St. Louis, MO, USA). Curcumin (purity 98%) was obtained from Tianjin Guangfu Fine Chemical Research Institute (Tianjin, China). All other reagents were of analytical grade.

### 2.2. Preparation of Carboxymethylated Short-Chain Amylose

Carboxymethylated short-chain amylose (CSA) was prepared following the procedure outlined by Ji et al. [18]. In brief, waxy corn starch underwent pullulanase treatment to eliminate branching, yielding short-chain amylose. The obtained short-chain amylose was subsequently frozen at −20 °C and subjected to freeze-drying. Next, a mixture of 90 mL of 90% ethanol solution and sodium hydroxide (2.57 g) was introduced into a three-neck flask, and thoroughly stirring ensured complete dissolution of sodium hydroxide. The freeze-dried short-chain amylose (10 g) was added to the aforementioned mixed solution, maintaining the temperature at 40 °C with continuous stirring for 1 h. A chloroacetic acid solution (dissolved in 10 mL of 90% ethanol) was gradually introduced within 10 min, and the reaction proceeded at 40 °C for 3 h. Following the reaction, the sodium hydroxide in the mixture was neutralized with hydrochloric acid (0.4 M), and the resulting precipitate underwent washing with a 95% ethanol solution until chloride-free (confirmed through the silver chloride test). The washed precipitate was then dried to yield CSA.

### 2.3. Preparation of Curcumin-Loaded zein@CSA Core–Shell Nanoparticles

**Figure 1:**
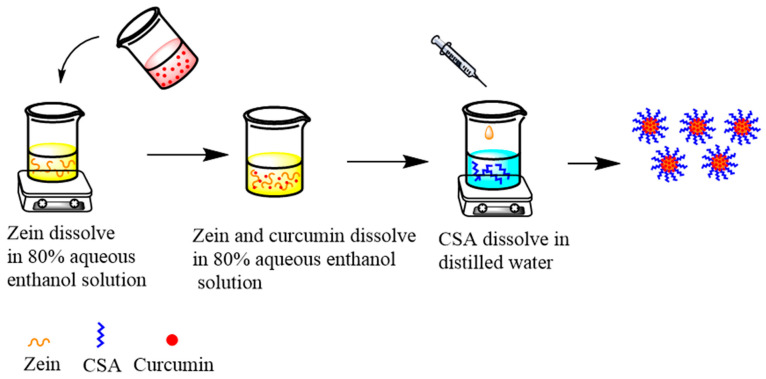
Flow chart of composite nanoparticles preparation.

The preparation of curcumin-loaded zein@CSA core–shell nanoparticles followed the anti-solvent co-precipitation method outlined by Ji et al. [18]. The flow chart is shown in Figure 1. In brief, 0.8 g of zein was dissolved in 80 mL of 80% ethanol solution and stirred overnight at 500 rpm/min to ensure complete dissolution. Concurrently, 0.1 g of curcumin was dissolved in 20 mL of an 80% ethanol solution and stirred at 300 rpm/min for 30 min. The curcumin solution was then dripped into the zein solution, resulting in concentrations of 1 mg/mL for curcumin and 8 mg/mL for zein. The pH of the mixture was adjusted to 4.

Various masses of CSA were dispersed in deionized water and gelatinized at 90 °C with stirring at 300 rpm/min for 30 min. The solutions were cooled to room temperature, yielding CSA concentrations of 0, 2, 4, 6, 8, and 10 mg/mL. Subsequently, 5 mL of the curcumin–zein solution was slowly injected into a beaker containing 20 mL of CSA solution using a syringe. The mixture was stirred at 300 rpm/min for 30 min to form core–shell nanoparticles. The resulting dispersion underwent centrifugation at 3500× *g* for 10 min to eliminate unencapsulated curcumin particles. The supernatant was collected and labeled zein@CSA_n_, where *n* represents the concentration of added CSA.

### 2.4. Size and Charge of zein@CSA Core–Shell Nanoparticles

The average particle size, polydispersity index (PDI), and zeta-potential of zein@CSA core–shell nanoparticles were measured using a dynamic light scattering instrument (Nano ZS 90, Malvern Instruments Ltd., Worcestershire, UK) at 25 °C. To mitigate multiple scattering effects and enhance experimental accuracy, the samples underwent dilution to an appropriate concentration using ultrapure water before the analysis.

### 2.5. Encapsulation Efficiency (EE) and Loading Capacity (LC) of Curcumin

The determination of curcumin encapsulation efficiency (EE) and loading capacity (LC) in zein@CSA core–shell nanoparticles followed the procedure outlined by Liu et al. [3] with minor adjustments. In summary, the freshly prepared suspension of core–shell nanoparticles underwent centrifugation at 3000× *g* for 30 min to eliminate unencapsulated curcumin. To achieve absorbance values within the range of 0.1 to 1.0 cm^−1^, the centrifuged suspension of core–shell nanoparticles was diluted with an 80% ethanol solution to an appropriate concentration. The absorbance of the core–shell nanoparticle solution at a wavelength of 419 nm was recorded using a UV spectrophotometer. The concentration of curcumin within the core–shell nanoparticles was then calculated using a fitted standard curve (y = 0.1394 × x − 0.0039). The EE and LC of curcumin were determined using the following formulas:

(1) $$EE\left(\%\right)=\frac{amount\;of\;encapsulated\;curcumin(mg)}{total\;cuecumin\;added(mg)}\times 100$$

(2) $$LC\left(\%\right)=\frac{amount\;of\;encapsulated\;curcumin(mg)}{total\;amount\;of\;complex(mg)}\times 100$$

### 2.6. Fourier-Transform Infrared Spectroscopy

To obtain the Fourier-transform infrared (FTIR) spectra, samples were analyzed using an FTIR spectrometer (Jasco Inc., Easton, MD, USA). Approximately 4–5 mg of freeze-dried sample powder was placed on the detector, and a scan measurement was performed over the wavenumber range of 4000 to 500 cm^−1^.

### 2.7. X-ray Diffraction

To assess crystalline structures of different samples, X-ray diffraction (XRD) patterns were captured using an X-ray diffractometer (D8-ADVANCE; Bruker, Karlsruhe, Germany) with conditions set at 25 mA, 40 kV, and a 2θ angle range spanning 4 to 55°.

### 2.8. Fluorescence Spectroscopy Analysis

Fluorescence spectra were obtained employing a fluorescence spectrophotometer (F-2700, Hitachi, Tokyo, Japan). The nanoparticle solutions mentioned earlier underwent dilution to an appropriate concentration using deionized water. Emission spectra were recorded within the 500 to 700 nm range, with an excitation wavelength of 420 nm. The excitation and emission slits were configured at 5 nm, and the scanning speed was established at 300 nm/min.

### 2.9. Transmission Electron Microscopy Analysis

The morphological features of zein nanoparticles and zein@CSA_1.0_ core–shell nanoparticles were examined through a transmission electron microscope (HT7700 TEM, Hitachi, Tokyo, Japan). The freshly prepared nanoparticle suspension underwent dilution to an appropriate concentration and was then deposited onto a carbon-coated copper grid to create the samples. Excess dispersion liquid was eliminated using filter paper, followed by freeze-drying treatment.

### 2.10. Ionic Stability

Different concentrations of NaCl solution (10, 50, and 100 mM) were mixed in equal proportions with the freshly prepared suspension of core–shell nanoparticles for 1 h to ensure thorough mixing [9]. The mixture was subsequently stored at room temperature for 24 h, following which the average particle size of the core–shell nanoparticles was determined using the method outlined in Section 2.4.

### 2.11. Release Properties

To replicate the in vivo release behavior of curcumin from zein nanoparticles and zein@CSA core–shell nanoparticles, simulated gastric fluid (SGF) and simulated intestinal fluid (SIF) were prepared following a previous study with slight modifications [20]. Initially, all formulations were mixed (1:1, *v*/*v*) with SGF and agitated at 37 °C for 2 h. Subsequently, the solutions were transferred to an equal volume of SIF and further incubated for an additional 4 h. At predetermined time points (30, 60, 90, 120, 150, 180, 210, and 240 min), samples were collected and underwent centrifugation at 13,000× *g* for 10 min. The absorbance of the supernatant at a wavelength of 419 nm was recorded using a UV spectrophotometer. The concentration of free curcumin in the supernatant was then calculated using a fitted standard curve (y = 0.1394 × x − 0.0039).

### 2.12. Antioxidant Activities

Antioxidant activities were assessed by the DPPH method [21]. Specifically, for the DPPH free radical scavenging activity assay, the sample was mixed with an equal volume of DPPH ethanol solution and allowed to react in the dark for 30 min. The absorbance was then measured at 517 nm using a multiple reader, and the scavenging activity of the DPPH radical was calculated based on the equation:

(3) $$DPPH-Radical\;Scavenging\;Activity\;(\%)=\frac{Absorbance\;of\;control-Absorbance\;of\;sample\;after\;30\;min}{Absorbance\;of\;control}\times 100$$

### 2.13. In Vitro Cytotoxicity Assays

The cell viability of zein@CSA core–shell nanoparticles against Caco-2 cells was assessed using the MTT assay following our previously reported method [4]. The Caco-2 cells were seeded in 96-well plates at a density of 1 × 10^4^ cells per well, cultured for 24 h, and then incubated with core–shell nanoparticles at different concentrations (125–1000 µg/mL) for 24 h at 37 °C. Following incubation, the medium was aspirated, and MTT was added for a 4 h incubation at 37 °C before UV-vis absorption measurements.

### 2.14. Statistical Analysis

All experiments were replicated at least thrice to determine the mean and standard deviation. Statistical analysis was carried out using SPSS 26.0 for Windows (SPSS Inc., Chicago, IL, USA), utilizing analysis of variance (ANOVA) to evaluate statistical differences. Post-hoc analysis was conducted using the Duncan post hoc test to identify significant differences, with a significance level set at *p* < 0.05.

## 3. Results and Discussion

### 3.1. Particle Size and Zeta-Potential Characteristics

**Figure 2:**
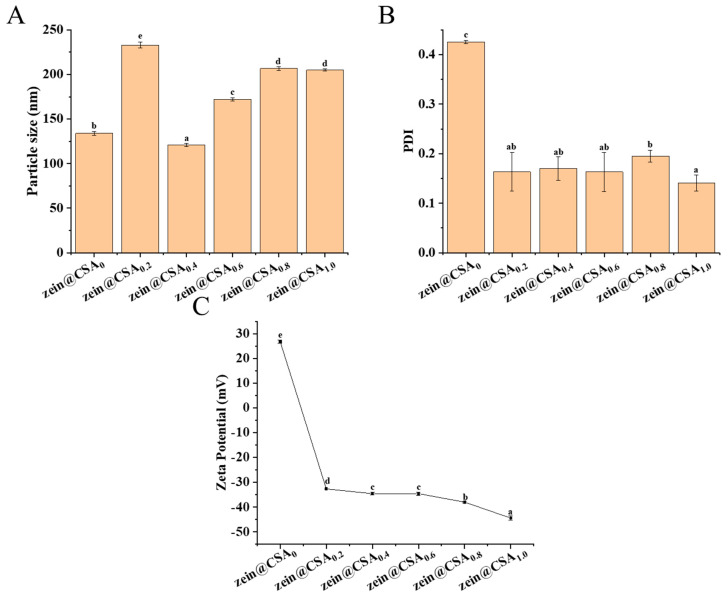
The prepared curcumin-loaded zein@CSA (zein@CSA_n_) nanoparticles: (**A**) mean particle diameter; (**B**) polydispersity index (PDI), and (**C**) zetapotential. Error bars are standard deviation (SD), the significant differences of different proportions are shown using lowercase letters (a–e) at a significant level of *p* < 0.05.

Figure 2 displays the particle size, PDI, and zeta-potential of the core–shell nanoparticles. In the absence of CSA, the particle size of the curcumin-loaded zein nanoparticles measured 133 ± 1.96 nm, with a PDI of 0.42 and a zeta-potential of +26.75 ± 0.64 mV. When the zein/CSA mass ratio was 4:1, the particle size of the zein@CSA_0.2_ core–shell nanoparticles increased to 232.97 ± 3.16 nm. This increase was attributed to the relatively low content of CSA at this ratio, which was insufficient to completely cover the surface of zein nanoparticles, leading to certain bridging flocculation. The zeta-potential of the zein@CSA_0.2_ nanoparticles was −32.65 mV, and with an increase in the mass ratio of CSA, the zeta-potential showed a further decreasing trend. These results indicated that negatively charged CSA was electrostatically attracted and encapsulated on the surface of zein nanoparticles. When the zein/CSA mass ratio was 2:1, the zein@CSA_0.4_ core–shell nanoparticles exhibited the relatively smallest particle size of 120.90 ± 2.00 nm. This phenomenon was due to sufficient CSA adsorbed on the surface of the zein nanoparticles through electrostatic interactions at this ratio, facilitating the formation of a dense core–shell structure and resulting in a smaller particle size. As the zein/CSA mass ratio further increased, excess CSA aggregated on the surface of the zein, leading to a gradual increase in the particle size of the core–shell nanoparticles. It was noteworthy that compared to nanoparticles without CSA (zein@CSA_0_), the nanoparticles with added CSA showed lower PDI values, indicating that the addition of CSA significantly improved the dispersion of the nanoparticles, consistent with the reported results of Li et al. [22].

### 3.2. Encapsulation Efficiency (EE) and Loading Capacity (LC)

Table 1 details the EE and LC of curcumin in zein@CSA core–shell nanoparticles. The EE for zein@CSA_0_ nanoparticles was recorded at 33.82%. Notably, core–shell nanoparticles with a zein/CSA mass ratio of 4:1 exhibited the highest EE and LC, surpassing those of nanoparticles composed solely of zein.

A significant increase in curcumin EE was observed, rising from 33.82% in zein@CSA_0_ core–shell nanoparticles to 96.47% in zein@CSA_1.0_, representing a noteworthy improvement over previous findings [13]. The significant enhancement of EE indicated that curcumin has a strong affinity for the delivery system, which may be attributed to the following reasons: firstly, the non-covalent interactions among curcumin, CSA, and zein significantly increased EE [23]; secondly, the synergistic effect of CSA and zein on curcumin encapsulation further improved EE [24]. Previous studies demonstrated that saponin could improve the EE of curcumin [24], and the incorporation of natural polymers was reported to boost EE in zein systems [25,26]. Interestingly, the LC for curcumin-loaded zein nanoparticles was lower than that in zein@CSA core–shell nanoparticles. This might be because, in the core–shell nanoparticles, the increased CSA content resulted in higher total complexation, leading to a decrease in the LC.

### 3.3. Fourier Transform Infrared Spectroscopy

**Figure 3:**
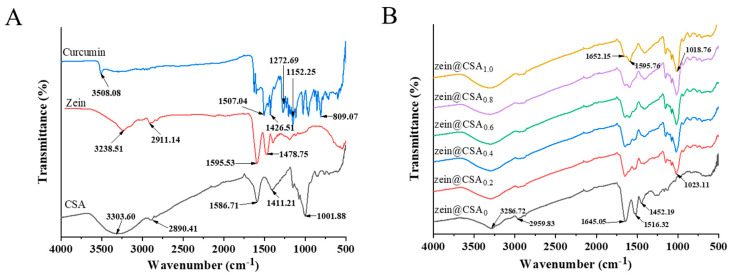
FTIR spectra of (**A**) curcumin, zein, CSA, and (**B**) zein@CSA_n_ nanoparticles.

The FTIR spectra of individual components (zein, CSA, and curcumin) and curcumin-loaded core–shell nanoparticles are depicted in Figure 3A,B. In the spectra of curcumin, characteristic peaks at 3510, 1629, 1606, 1504, 1287, and 1027 cm^−1^ were observed, corresponding to –OH stretching, C=C and C=O tensile vibration, C–O and C–C vibration, aromatic C–O stretching vibration, and C–O–C stretching vibration, respectively. Notably, the distinctive curcumin peak was nearly absent in the core–shell nanoparticles, indicating the successful encapsulation of curcumin. This observation aligns with the findings of Liu et al., who reported that when curcumin was incorporated into nanoparticles, the peaks observed for pure curcumin disappeared [3].

Individual zein and CSA exhibited characteristic peaks at 3238.5 and 3303.6 cm^−1^, respectively, in the 3100–3500 cm^−1^ range, indicating the stretching vibration of hydroxyl groups (O–H). In the core–shell nanoparticles, these O-H group peaks shifted to 3286 cm^−1^ (0.2%), 3294 cm^−1^ (0.4%), 3305 cm^−1^ (0.6%), 3316 cm^−1^ (0.8%), and 3320 cm^−1^ (1.0%), suggesting the formation of hydrogen bonds between zein and CSA (Figure 3B). Similar results have been reported by Du et al., who observed a similar hydrogen bond in soluble soybean polysaccharide/zein complex nanoparticles [27]. Zein also exhibited characteristic peaks at 2911.1 cm^−1^ (C–H vibration) and in the 1450–1600 cm^−1^ range (amide I and amide II vibrations). After forming core–shell nanoparticles, these amide bands shifted to 1645.0 cm^−1^ and 1516.3 cm^−1^, indicating hydrophobic and electrostatic interactions between zein and CSA, as reported in previous studies by Ye et al. [28].

In summary, non-covalent interactions, encompassing hydrophobic, hydrogen bonding, and electrostatic interactions, were likely present among curcumin, zein, and CSA. Similar findings were reported by Chang et al. [29], who stated that the formation of casein–zein–polysaccharide nanoparticles was driven by hydrogen bonds and hydrophobic interactions between them, consistent with previous research findings [26].

### 3.4. XRD

**Figure 4:**
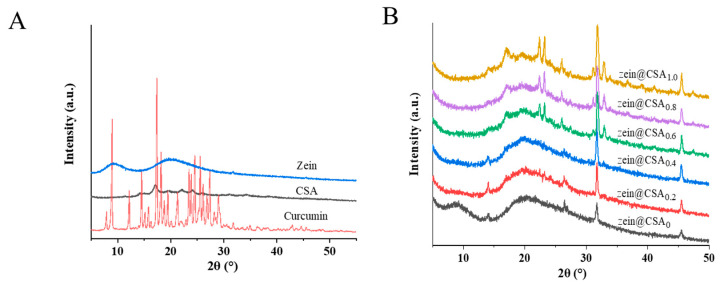
XRD patterns of (**A**) curcumin, zein, CSA, and (**B**) zein@CSA_n_ nanoparticles.

The X-ray diffraction (XRD) patterns of curcumin, zein, CSA, and curcumin-loaded core–shell nanoparticles are illustrated in Figure 4A,B. The XRD patterns of pure curcumin exhibited a crystalline structure, evidenced by peaks at 8.90°, 12.16°, 14.51°, 17.36°, 23.44°, 24.53°, and 25.5°. Zein displayed two broad peaks at about 9° and 19°, indicative of the amorphous nature of native proteins. After the formation of zein@CSA core–shell nanoparticles, the characteristic peak of zein at 9° vanished, confirming that CSA was effectively coated on the surface of zein nanoparticles. The characteristic peaks of curcumin in zein@CSA_0_ and zein@CSA core–shell nanoparticles disappeared, indicating the successful encapsulation of curcumin into the nanoparticles, manifested as an amorphous structure.

At lower CSA concentrations (0, 0.2%, and 0.4%), characteristic peaks appeared at a diffraction angle of 14°. However, with higher CSA concentrations (0.6%, 0.8%, and 1.0%), the peak at 14° vanished, replaced by new peaks at 17°, 22°, and 23°. This suggested that the interaction among curcumin, zein, and CSA led to the formation of non-crystalline complexes, confirming the successful encapsulation of curcumin within the core–shell nanoparticles. As the CSA concentration increased, the aggregation and induced chain binding of protein molecules led to the disappearance of the original peak at 14°. These results indicated that the interaction among curcumin, zein, and CSA, along with the dispersion of curcumin in an amorphous form within the core–shell nanoparticles, laid the foundation for the effective release of curcumin. Previous studies by Patel et al. and Dai et al. have demonstrated that encapsulating curcumin in nano-composites can inhibit its crystallization [23,30]. Additionally, Li et al. reported that compared to its crystalline form, amorphous curcumin is more readily released from nanoparticles [31].

### 3.5. Fluorescence Spectroscopy

**Figure 5:**
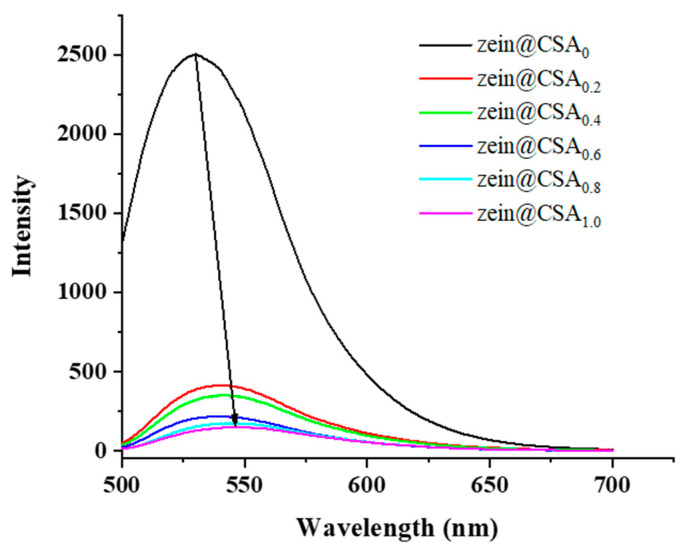
Fluorescence spectra of zein@CSA_n_ nanoparticles.

The fluorescence spectra of curcumin-loaded core–shell nanoparticles are depicted in Figure 5. The zein nanoparticles exhibited a pronounced fluorescence emission peak at 529.5 nm. With the addition of CSA to the zein nanoparticles, there was a noticeable decrease in the fluorescence intensity of this peak with increasing CSA concentrations, and the peak blue-shifted to a wavelength of around 548.5 nm. This shift suggests a change in the polarity of the tryptophan residue microenvironment. These findings were consistent with previous research, indicating that the introduction of CSA led to fluorescence quenching in zein nanoparticles due to non-covalent interactions between zein and CSA [18].

### 3.6. Transmission Electron Microscopy

**Figure 6:**
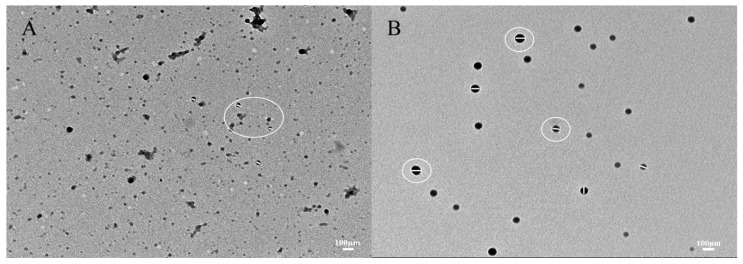
TEM images of (**A**) zein@CSA_0_ nanoparticles and (**B**) zein@CSA_1.0_ nanoparticles.

Figure 6A,B illustrates the microstructure of zein and zein@CSA_1.0_ core–shell nanoparticles. The curcumin-loaded zein nanoparticles, characterized by irregular shapes, due to protein particle interactions during solvent evaporation, formed clusters. In contrast, the zein@CSA_1.0_ core–shell nanoparticles exhibited a consistently spherical shape, evenly dispersed. These results highlight that the hydrophilic CSA surface coating on the zein nanoparticles, offering electrostatic and hydrophobic interactions, contributes to a more compact structure. This aligns with Zhang et al.’s findings, which indicated similar aggregation in zein particles when insufficient sodium caseinate was applied to the surface of zein nanoparticles [32].

### 3.7. Ionic Stability Analysis

**Figure 7:**
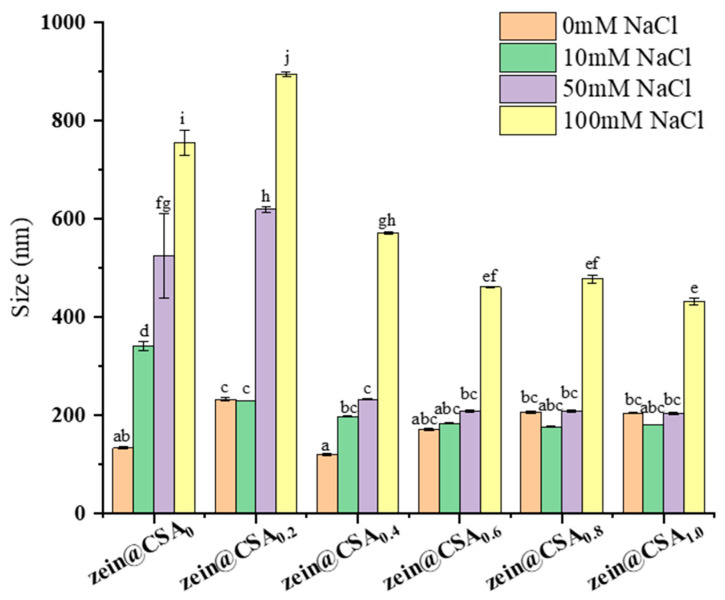
Effect of different NaCl concentrations (0 mM, 10 mM, 50 mM, and 100 mM) on the particle size of nanoparticles. (a–j) said the difference was statistically significant (*p* < 0.05).

Figure 7 depicts the influence of varying NaCl concentrations (0, 10, 50, and 100 mM) on the particle size of both zein nanoparticles (zein@CSA_0_) and zein@CSA core–shell nanoparticles. The introduction of NaCl solution significantly increased the particle size of zein nanoparticles, indicating their instability under these solutions. Conversely, with an elevated CSA concentration, the ionic stability of zein@CSA core–shell nanoparticles showed marked improvement (*p* < 0.05). In the NaCl concentration range of 10–50 mM, the particle sizes of zein@CSA_0.4_, zein@CSA_0.6_, zein@CSA_0.8_, and zein@CSA_1.0_ core–shell nanoparticles remained relatively stable, possibly attributed to the presence of the CSA “shell” structure, which imparted stability within this concentration range. With increasing amounts of CSA added, the CSA shell of the core–shell nanoparticles became thicker, thereby enhancing the protective effect on the nanoparticles. Consequently, low NaCl concentrations failed to disrupt the interaction between the CSA shell and the zein core. However, at an increased ionic strength to 100 mM, all nanoparticles experienced size augmentation. This outcome may be attributed to the addition of NaCl solution, weakening non-covalent interactions between CSA and zein through electrostatic screening, thereby resulting in loose internal connections among zein@CSA core–shell nanoparticles.

### 3.8. Antioxidant Activity

**Figure 8:**
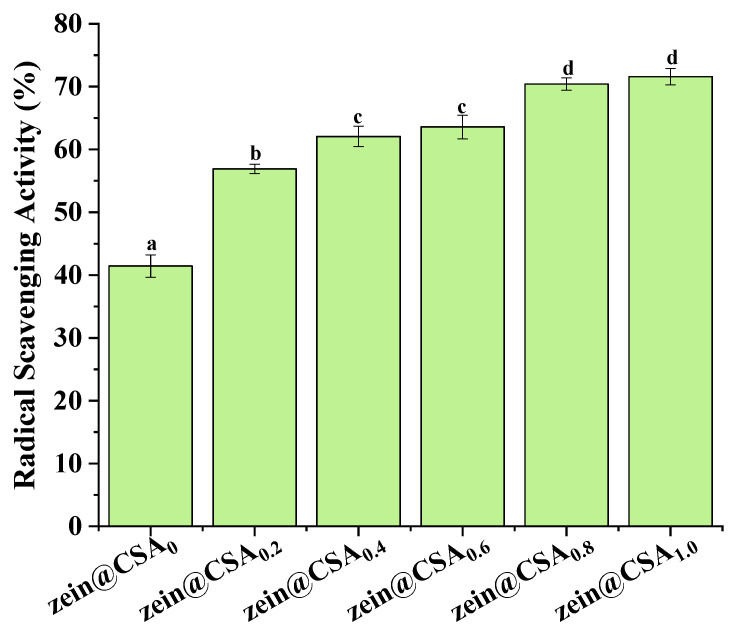
DPPH·scavenging capacity of zein@CSA_n_ nanoparticles. (a–d) said the difference was statistically significant (*p* < 0.05).

Figure 8 illustrates the impact of encapsulation on the DPPH radical scavenging capacity of curcumin. The antioxidant activity of curcumin within zein@CSA core–shell nanoparticles significantly increased with the rise in the CSA concentration, with zein@CSA_1.0_ core–shell nanoparticles exhibiting the highest DPPH scavenging capacity (71.5%). This enhancement could be attributed to the improved water solubility of curcumin within the hydrophilic zein@CSA core–shell nanoparticles. This improvement facilitated more effective interactions between free radicals and curcumin in the aqueous phase, leading to the elimination of free radicals and an increased antioxidant capacity of curcumin. This finding aligns with previous studies, indicating a substantial boost in the antioxidant capacity of curcumin when encapsulated in zein@CSA core–shell nanoparticles [31,33]. Additionally, other studies demonstrated that encapsulation in various delivery systems, such as thiol-modified hyaluronic acid–zein [14], soybean protein–dextran dialdehyde nanocomplexes [34], whey protein isolate–short linear glucan nanoparticles [35], and fucoidan–zein nanoparticles [36], also significantly improved the antioxidant capacity of curcumin.

### 3.9. Release Behavior of the Nanoparticles

**Figure 9:**
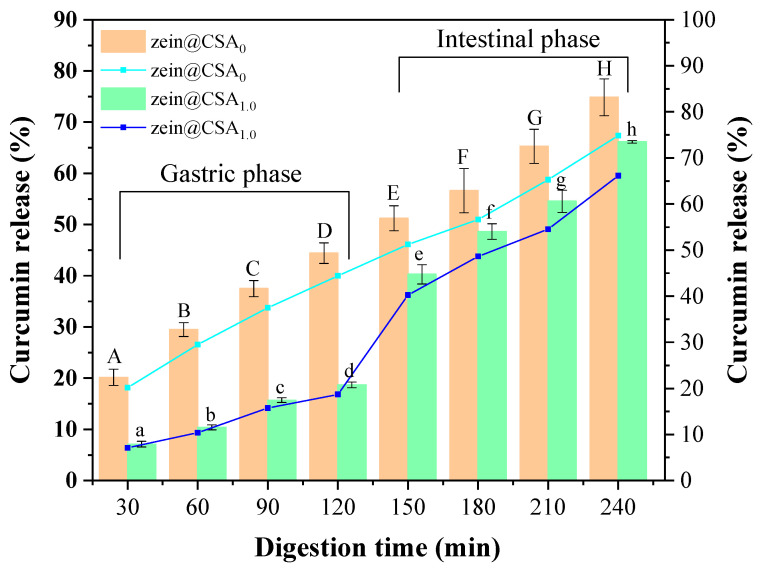
Release profiles of curcumin from zein@CSA_n_ nanoparticles during in vitro simulated gastrointestinal conditions (zein@CSA_0_ nanoparticles and zein@CSA_1.0_ nanoparticles). (A–H) indicates that zein@CSA_0_ was significantly different at different digestion times (*p* < 0.05) and (a–h) indicates that zein@CSA_1.0_ was significantly different at different digestion times (*p* < 0.05).

Figure 9 depicts the curcumin release profiles of zein nanoparticles and zein@CSA_1.0_ core–shell nanoparticles in the gastrointestinal tract. With an increased digestion time, the cumulative release rate of curcumin in both zein nanoparticles and zein@CSA_1.0_ core–shell nanoparticles gradually rose. After 120 min of simulated gastric fluid digestion, the release rates of curcumin from zein nanoparticles and zein@CSA_1.0_ core–shell nanoparticles were 44.41% and 18.68%, respectively. Notably, the release rate of curcumin in zein@CSA_1.0_ core–shell nanoparticles was lower than that of zein nanoparticles. This signifies that, encapsulated in the zein@CSA_1.0_ core–shell nanoparticles, curcumin could be effectively preserved during the simulated gastric juice stage. This might be attributed to the absence of starch-hydrolyzing enzymes in the stomach, leading to the strong resistance of the CSA shell to gastric protease hydrolysis. Consequently, zein@CSA core–shell nanoparticles effectively protected curcumin from gastric effects and released it into the small intestine [18].

During the small intestinal digestion stage, both zein nanoparticles and zein@CSA_1.0_ core–shell nanoparticles demonstrated the rapid release of curcumin. The rapid release of curcumin during the small intestinal digestion stage might be attributed to the strong hydrolytic capacity of pancreatic protease on the remaining zein after gastric phase hydrolysis, facilitating the release of curcumin from the core–shell nanoparticles in the small intestine [37,38]. Additionally, according to previous studies, pH might affect the charge density of CSA, altering the structure of the “shell”. As the pH increased during the small intestinal digestion stage, the deprotonation of the carboxylic group of CSA to the charged species (–COO^−^) caused the “shell” to become loose, thereby increasing the release rate of curcumin [39]. After 240 min of digestion, the release rate of curcumin from zein@CSA_1.0_ core–shell nanoparticles was still lower than that from zein nanoparticles. These results suggested that the release effect of zein@CSA_1.0_ core–shell nanoparticles in the small intestinal digestion stage was more sustained than that of zein nanoparticles. This more sustained controlled-release behavior might be attributed to the addition of the CSA surface layer to the zein@CSA core–shell nanoparticles, providing a better barrier for curcumin release and delaying the release rate of curcumin, consistent with the results of Ji et al. [4].

### 3.10. In Vitro Cytotoxicity

**Figure 10:**
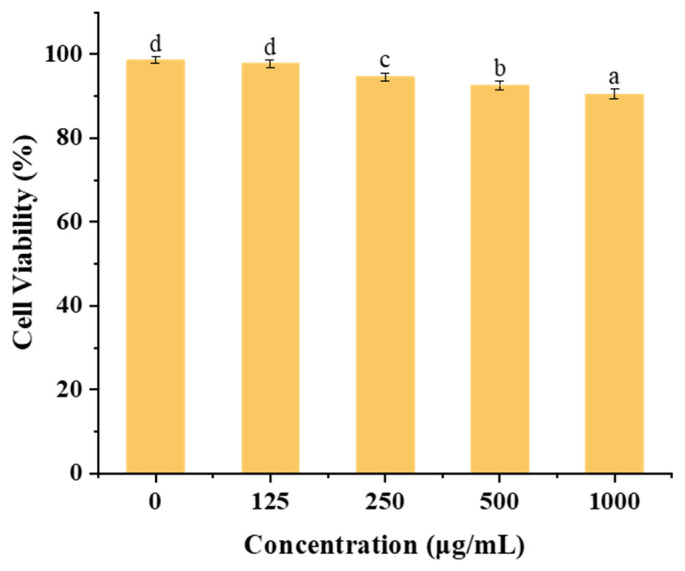
In vitro cell viability of Caco-2 cells against zein@CSA_1.0_ nanoparticles with different concentrations. Note: columns marked with different letters denote significant difference at *p* < 0.05.

Figure 10 illustrates the impact of varying concentrations (125–1000 µg/mL) of zein@CSA_1.0_ core–shell nanoparticles on cytotoxicity in Caco-2 cells. Within the 0 to 500 μg/mL concentration range, the zein@CSA_1.0_ core–shell nanoparticles demonstrated minimal cytotoxicity after 24 h incubation, maintaining cell viability above 92%. Even at a higher concentration of 1000 µg/mL, after 48 h of incubation, the zein@CSA_1.0_ core–shell nanoparticles sustained cell viability at 90.50%. These results indicate that the core–shell nanoparticles exhibited low cytotoxicity and enhanced biocompatibility at the tested concentrations.

## 4. Conclusions

Zein@CSA core–shell nanoparticles, employing zein and CSA, were successfully synthesized through anti-solvent precipitation for the effective delivery of curcumin. These nanoparticles, featuring a consistently spherical shape and high dispersity, were formed via the self-assembly properties of zein. The creation of these core–shell nanoparticles was primarily driven by hydrogen bonding, hydrophobic interactions, and electrostatic forces. The zein@CSA core–shell nanoparticles exhibited notable ionic stability and encapsulation efficiency (EE), accompanied by a delayed curcumin release and enhanced antioxidant capacity. Additionally, within the tested concentration range of 125–1000 μg/mL, the nanoparticles demonstrated negligible cytotoxicity to Caco-2 cells. In summary, the “core-shell” nanoparticles formed by zein–CSA not only address the aggregation issue associated with zein as a standalone encapsulation carrier, but also enhance the encapsulation efficiency and stability of curcumin. Therefore, given the antimicrobial properties of curcumin, the core–shell nanoparticles could hold potential applications in food safety and preservation. Meanwhile, this study holds significant practical implications for the loading and sustained release of other active compounds, serving as an efficient system for the encapsulating, delivering, and controlled release of active substances into functional foods.

## Figures and Tables

**Table 1:** EE and LC of zein@CSA_n_ complex nanoparticles.

Sample	EE (%)	LC (%)
zein@CSA_0_	33.82 ± 3.27 ^a^	3.76 ± 0.36 ^a^
zein@CSA_0.2_	91.47 ± 3.86 ^b^	8.32 ± 0.35 ^d^
zein@CSA_0.4_	91.19 ± 8.49 ^b^	7.01 ± 0.65 ^c^
zein@CSA_0.6_	91.19 ± 8.21 ^b^	6.08 ± 0.55 ^bc^
zein@CSA_0.8_	92.20 ± 1.45 ^b^	5.42 ± 0.09 ^b^
zein@CSA_1.0_	96.47 ± 1.52 ^b^	5.09 ± 0.08 ^b^

Results are expressed as mean ± standard deviation (*n* = 3). Different letters in the same column indicate significant differences (*p* < 0.05).

## Data Availability

The original contributions presented in the study are included in the article, further inquiries can be directed to the corresponding author.
